# Lymphatic endothelial cells attenuate inflammation via suppression of dendritic cell maturation

**DOI:** 10.18632/oncotarget.9820

**Published:** 2016-06-05

**Authors:** Ailsa J. Christiansen, Lothar C. Dieterich, Isabel Ohs, Samia B. Bachmann, Roberta Bianchi, Steven T. Proulx, Maija Hollmén, David Aebischer, Michael Detmar

**Affiliations:** ^1^ Institute of Pharmaceutical Sciences, Swiss Federal Institute of Technology, ETH Zurich, Zurich, Switzerland

**Keywords:** inflammation, lymphangiogenesis, VEGF-C, Immunology and Microbiology Section, Immune response, Immunity

## Abstract

Vascular endothelial growth factor-C (VEGF-C)-induced lymphangiogenesis and increased tissue drainage have been reported to inhibit acute and chronic inflammation, and an activated lymphatic endothelium might mediate peripheral tolerance. Using transgenic mice overexpressing VEGF-C in the skin, we found that under inflammatory conditions, VEGF-C-mediated expansion of the cutaneous lymphatic network establishes an immune-inhibitory microenvironment characterised by increased regulatory T (Treg) cells, immature CD11c+CD11b+ dendritic cells (DCs) and CD8+ cells exhibiting decreased effector function. Strikingly, lymphatic endothelial cell (LEC)-conditioned media (CM) potently suppress DC maturation with reduced expression of MHCII, CD40, and IL-6, and increased IL-10 and CCL2 expression. We identify an imbalance in prostaglandin synthase expression after LEC activation, favoring anti-inflammatory prostacyclin synthesis. Importantly, blockade of LEC prostaglandin synthesis partially restores DC maturity. LECs also produce TGF-β1, contributing to the immune-inhibitory microenvironment. This study identifies novel mechanisms by which the lymphatic endothelium modulates cellular immune responses to limit inflammation.

## INTRODUCTION

The lymphatic vascular system plays a key role in the transport of interstitial fluid, antigens and immune cells from the periphery to lymph nodes where adaptive immune responses are generated. Expansion of the lymphatic network is observed in numerous inflammatory conditions [1–3], and transgenic overexpression or intradermal injection of the lymphangiogenic factor vascular endothelial growth factor (VEGF)-C inhibits acute and chronic skin inflammation [4–8]. Similarly, viral VEGF-C administration reduced joint lesion severity in chronically inflamed arthritic joints [9] and provided protection against experimental inflammatory bowel disease [10], whereas blockade of VEGFR3 signaling aggravated inflammatory bowel disease [11]. Enhanced lymphatic drainage function has been implicated as the primary mechanism underlying these potent anti-inflammatory effects of VEGF-C. However, VEGF-C may also directly attenuate cellular immunity *via* regulation of macrophage plasticity and activation [10, 12, 13]. Moreover, activated lymphatic endothelial cells (LECs) are involved in the induction of peripheral tolerance [14–18] and might play a role in the generation of an immunotolerant tumor microenvironment [19].

In the present study, we investigated if VEGF-C regulates cellular immunity in cutaneous inflammation, and whether it acts directly on inflammatory cells or indirectly *via* activation and expansion of the lymphatic endothelium, using K14-VEGF-C transgenic mice that express human VEGF-C in the skin under control of the keratin-14 promoter [20]. These mice have an expansion of lymphatic but not blood vessels in the skin [20] and show reduced inflammation during chemical skin carcinogenesis [21], acute bacterial pathogen-induced skin inflammation [8], in response to UVB irradiation, and in oxazolone-induced delayed-type hypersensensitivity reactions [5]. We used the PKC activator 12-O-tetradecanoylphorbol-13-acetate (TPA) to induce chronic skin inflammation. This was based on its ability to induce epidermal hyperplasia [22, 23] and enhance the K14-promoter driven transgene expression [21, 24, 25]. We found that VEGF-C-mediated expansion of the lymphatic network establishes an immune-inhibitory cutaneous microenvironment. VEGF-C had no direct effects on dendritic cell (DC) maturation but LEC-conditioned media (CM) potently suppressed DC maturation, which was partially restored upon blockade of LEC prostaglandin synthesis. This study identifies a new mechanism by which the expanded lymphatic vasculature modulates cellular immune responses and limits inflammation.

## RESULTS

### Reduced antigen-presentation capacity in the inflamed skin of VEGFC transgenic mice

Skin lysates from K14-VEGFC mice contained VEGF-C protein (Supplementary Figure 1A) whose levels were strongly increased under inflammatory conditions, confirming efficient transgene expression in the skin. VEGF-C levels were also higher in the sera of uninflamed and inflamed K14-VEGFC mice than in wildtype (WT) littermate controls (Supplementary Figure 1B). The lymphatic network in the normal and inflamed skin of K14-VEGFC mice was significantly expanded, as determined by staining for the lymphatic specific marker LYVE-1 (Supplementary Figure 1C and 1D), which confirmed that the transgenic VEGF-C was biologically active. Although dilated, lymphatic vessels in K14-VEGFC mice contained button-type junctions that were similar to those observed in wildtype mice when co-stained for LYVE-1 and VE-cadherin (Supplementary Figure 1E).

**Figure 1:**
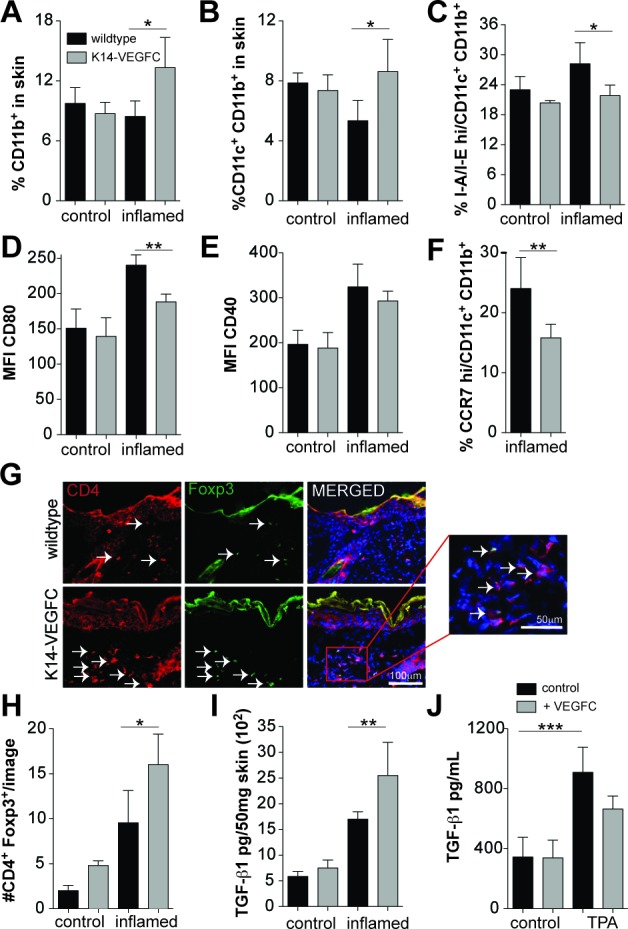
Inflamed skin of K14-VEGFC mice has elevated numbers of immature CD11c+CD11b+ cells and increased proportions of regulatory T cells Flow cytometry was used to determine the proportions of CD11b+ (**A**) and CD11c+CD11b+ (**B**)cells in the skin of control (*n* = 3 per genotype) and inflamed (*n* = 4 per genotype) wildtype and K14-VEGFC mice. CD11c+CD11b+ cells were also assessed for their expression of I-A/I-E (MHCII) (**C**), CD80 (**D**), CD40 (**E**) and CCR7 (**F**) (*n* = 4 per genotype/treatment except *n* = 7 for CCR7 in inflamed K14-VEGFC). Skin sections from wildtype and K14-VEFC control (*n* = 3 per genotype) and inflamed (*n* = 4 per genotype) mice were co-stained for CD4 and Foxp3. Representative fluorescent images for inflamed skin are shown in (**G**) (Scale bar: 100 μm). Left panels: CD4 (red); middle panels: Foxp3 (green); right panels: merged image of CD4, Foxp3, and Hoechst (blue) to visualize nuclei. The insert is a magnified region of the merged image as indicated (scale bar: 50 μm). Arrows indicate Foxp3+CD4+ cells. Foxp3+CD4+ cells per image were quantified and are shown in (**H**) (control *n* = 3, inflamed *n* = 4). Black bars: wildtype mice. Grey bars: K14-VEGFC mice. TGF-β1 protein levels were quantified in the back skin of control and inflamed wildtype (black bars) and K14-VEGFC mice (grey bars) (*n* = 4 per treatment and genotype) by ELISA (**I**) TGF-β1 protein levels were also quantified in cell culture supernatants taken from TPA (20 ng/mL) and recombinant VEGF-C (500 ng/mL) treated lymphatic endothelial cells (**J**) For all graphs, data shown are the mean ± SD. Two-way ANOVA with Bonferroni post-test was used to assess statistical significance except (F) where Student's t-test was applied. *p < 0.05,**p < 0.01.

We next investigated the effects of VEGF-C overexpression on the immune cell infiltrates in inflamed skin. No differences in the proportions of CD11b+ cells were detected in the normal skin of K14-VEGFC mice (Figure 1A), whereas these mice had elevated numbers of CD11b+ cells under inflammatory conditions (Figure 1A). This was predominantly due to a significant increase in the CD11c+CD11b+ DC population (Figure 1B). A slight, but not significant increase in CD11b+/F4/80+ macrophages and CD11b+/Gr-1+ myeloid derived suppressor cells was also observed (Supplementary Figure 1F-1G).

We next examined the effects of VEGF-C overexpression on DC subpopulations. No differences in the proportions of CD11c+CD11b+ cells expressing high levels of MHCII invariant chain I-A/I-E were observed between uninflamed WT mice and K14-VEGFC mice, as assessed by flow cytometry (Figure 1C). Under inflammatory conditions however, significantly fewer CD11c+CD11b+ cells expressed high levels of I-A/I-E in the skin of K14-VEGFC mice (Figure 1C). Similarly, in the absence of inflammation, no significant differences in the levels of co-stimulatory CD80 or CD40 were observed in CD11c+CD11b+ I-A/I-E^hi^ cells (Figure 1D-1E), whereas under inflammatory conditions, the CD11c+CD11b+ I-A/I-E^hi^ cells in the skin of K14-VEGFC mice had significantly reduced levels of co-stimulatory CD80 (Figure 1D). CD40 expression was also slightly reduced, however this was not statistically significant (Figure 1E). Importantly, CD11c+CD11b+ cells in the skin of inflamed K14-VEGFC mice expressed significantly lower levels of CCR7 (Figure 1F), a key chemokine receptor implicated in DC migration to draining lymph nodes [26].

### Inflamed skin of K14-VEGFC mice has elevated regulatory T cell numbers

Immature dendritic cells have the ability to prime naive T cells to differentiate into Treg cells [27, 28]. Using immunofluorescence stains and image quantification, we found significantly increased numbers of both CD4+ and CD8+ cells in inflamed skin when compared to normal skin (Supplementary Figure 1H-1I), but no significant differences were observed in WT *versus* K14-VEGFC mice. The numbers of immunosuppressive Tregs, quantified *via* CD4+Foxp3+ co-staining (Figure 1G), were significantly higher in K14-VEGFC mice than in WT mice (Figure 1H) in inflamed skin. No significant differences in the proportions of CD4+, CD8+ or CD4+Foxp3+ cells were observed in the thymus (CD4+, CD8+, CD4+CD8+ or CD4+Foxp3), spleen, LNs and blood (CD4+, CD8+, or CD4+Foxp3), when comparing K14-VEGFC mice with WT controls (Supplementary Figure 2A-2E), indicating that the differences in the proportions of Foxp3+ cells in the skin are due to a local effect and not a result of systemic differences between transgenic and WT mice.

**Figure 2:**
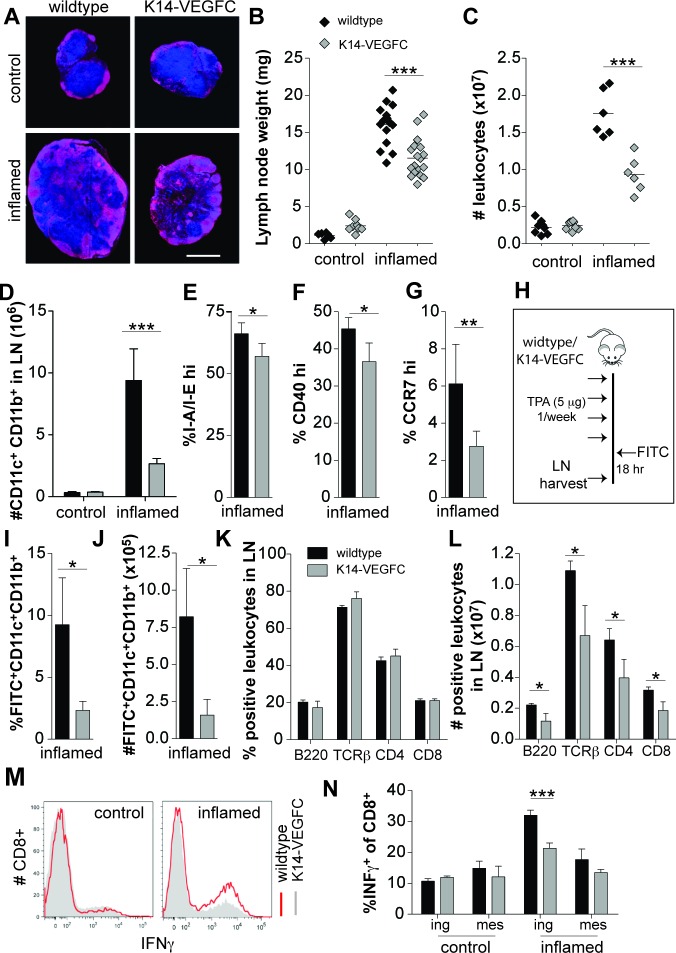
A reduced inflammatory response is observed in skin-draining inguinal LNs of K14-VEGFC mice Skin-draining inguinal LNs of untreated and inflamed wildtype and K14-VEGFC mice were harvested. Central transverse sections of inguinal LNs were stained for B220 (red) and Hoechst (blue). Representative immunofluorescent images are shown in (**A**) (Scale bar: 1 mm). LNs were weighed (**B**) and the cellularity determined using a hemocytometer (**C**) Each data point represents a single lymph node. The horizontal line represents the mean. Inguinal LNs from untreated (*n* = 3 per genotype) and inflamed (*n* = 4 per genotype) mice were analysed for their numbers of CD11c^+^CD11b^+^ cells using flow cytometry (**D**) Proportions of I-A/I-E^hi^ (**E**), co-stimulatory CD40^hi^ (**F**) and CCR7^hi^(**G**) cells were also quantified on CD11c^+^CD11b^+^ cells within inflamed inguinal LNs (*n* = 4, 4, and 7, respectively). Data are presented as mean ± SD. To investigate the ability of CD11c^+^CD11b^+^ cells to migrate from inflamed skin to skin-draining LNs, 5% FITC thioglycolate/acetone 1:1 v/v was applied to the inflamed backskin of wildtype (*n* = 4) and K14-VEGFC mice (*n* = 4) see schematic (**H**) The proportions of FITC-positive CD11c+CD11b+ cells in the draining LNs (axillary + inguinal) were quantified by flow cytometry (**I**) and the total number of migrating cells calculated (**J**) Proportions of B220, TCRβ, CD4 and CD8 positive cells were quantified using flow cytometry in skin-draining inguinal LNs from inflamed wildtype (*n* = 3) and K14-VEGFC (*n* = 3) mice (**K**) Total cellularity in the lymph node for each marker was also quantified and is shown in (**L**) Leukocytes isolated from inguinal and mesenteric LNs were stimulated with PMA/ionomycin and analysed for IFN-γ-production. Representative flow cytometry histograms are shown (**M**) Proportions of IFN-γ^+^CD8^+^ T cells in leukocytes isolated from control and inflamed mice are shown (**N**) (*n* = 4 per treatment and genotype). All data are presented as mean ± SD. Figures B-D were analysed using Two-way ANOVA with Bonferroni's post-test to assess statistical significance. Student's t-test was applied to all other figures. *p < 0.05,**p < 0.01 ***p < 0.001.

TGF-β1 is a potent immunomodulatory cytokine implicated in the generation of tolerogenic dendritic cells [29, 30] and is a critical factor in both the generation and maintenance of Tregs [31, 32], and their effector function [33, 34]. In untreated skin, no significant differences in TGF-β1 protein levels, determined by ELISA, were detected (Figure 1I), whereas after a single treatment with TPA, TGF-β1 was significantly upregulated in the skin of K14-VEGFC mice (Figure 1I). Cultured LECs produced relatively high levels of TGF-β1, as determined by ELISA of cell culture supernatants (345.0±132.0 pg/mL) (Figure 1J). TGF-β1 was not significantly regulated by VEGF-C directly; however, it was significantly increased nearly 3 fold upon stimulation with TPA (909.3±167.2 pg/mL) (Figure 1J), suggesting that the expanded and activated lymphatic endothelium in VEGFC transgenic mice contributed to the tolerogenic milieu *via* production of TGF-β1.

### Skin-draining lymph nodes of inflamed K14-VEGFC mice are reduced in size, cellularity and effector cell activity

The inguinal LNs of inflamed K14-VEGFC mice were significantly smaller than those of inflamed WT mice (Figure 2A and 2B). The leukocyte cellularity was also significantly reduced (Figure 2C), indicating that the inflammatory response was attenuated. Using flow cytometry, a significant reduction in the number of CD11c+CD11b+ cells was observed in the skin draining LNs of inflamed, but not uninflamed, K14-VEGFC mice (Figure 2D). Reflecting the findings in the skin, CD11c+CD11b+ cells in draining LNs of inflamed K14-VEGFC mice expressed significantly lower levels of I-A/I-E, costimulatory CD40 and CCR7 (Figure 2E, 2F and 2G).

**Figure 3:**
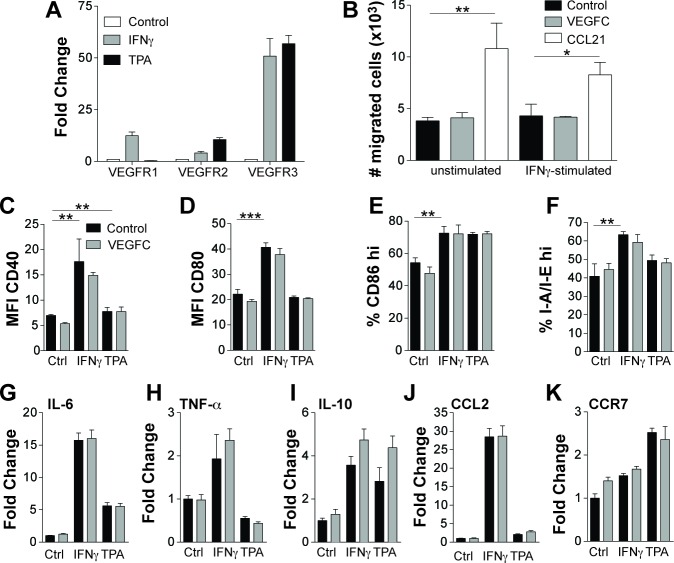
VEGF-C does not directly modulate the phenotype of CD11c+ cells Bone marrow derived CD11c+ cells were stimulated *in vitro* for 24 hours with IFN-γ (100 ng/mL) or TPA (10 ng/mL) and assessed for VEGFR expression using quantitative RT-PCR (**A**) Data are shown as fold change relative to control treated cells. Control and IFN-γ-stimulated bone marrow derived CD11c+ cells were assessed for their ability to migrate towards VEGF-C (500 ng/mL) in an *in vitro* transwell migration assay (**B**) The positive control of dendritic cell migration towards CCL21 (100 ng/mL) is also shown. Mean number of cells migrated per well (*n* = 3) is shown ± SD. Bone marrow derived CD11c+ cells were stimulated for 24 hours *in vitro* with IFN-γ (100 ng/mL) or TPA (10 ng/mL) in the presence or absence of VEGF-C (500 ng/mL) and were assessed for their expression of CD40 (**C**), CD80 (**D**), CD86 (**E**) and I-A/I-E (**F**) using flow cytometry. Data are shown as mean ± SD. No statistically significant differences were observed when comparing control to VEGF-C treated cells with or without IFN-γ or TPA stimulation. mRNA isolated from likewise treated cells were assessed using quantitative real-time PCR for the expression of IL-6 (**G**), TNF-α (**H**), IL-10 (**I**), CCL2 (**J**) and CCR7 (**K**). Data are shown as the fold change relative to control treated cells. Student's t-test was used to assess statistical significance (Figures B-F). *p < 0.05, **p < 0.01 ***p < 0.001.

We next investigated the ability of CD11c+CD11b+ cells to migrate from inflamed skin to the skin-draining LNs (schematic shown in Figure 2H). Following dermal FITC painting, the skin draining LNs of K14-VEGFC mice contained significantly fewer FITC+CD11c+CD11b+ cells that had migrated from the inflamed dermis (Figure 2I and 2J). This reduced migration most likely resulted from the observed decreased expression of CCR7 on CD11c+CD11b+ cells. Alternatively, a deregulated CCL21 gradient may also disrupt effective DC trafficking to draining LNs. We found that uninflamed and TPA-treated (single application) skin of K14-VEGFC mice had significantly higher levels of CCL21 than WT controls, and TPA-treatment significantly increased CCL21 expression (Supplementary Figure 3A), indicating that the normal tissue distribution of CCL21 might be altered in K14-VEGFC mice. We also observed increased dermal tissue clearance of a lymphatic specific tracer, with a reduced tissue half-life time in K14-VEGFC mice when compared to wildtype mice (Supplementary Figure 3B). This indicates that VEGF-C and the resulting expanded lymphatic vasculature led to increased lymph flow, which suggests that the observed reduced migration of DCs to draining LNs is not due to decreased lymphatic function in these mice. Increased flow from inflamed skin could however impact upon the dermal inflammatory milieu, for example by increasing the drainage of inflammatory molecules.

### The inflammatory response in skin draining LNs is reduced in K14-VEGFC mice

We next investigated other inflammatory cell populations within inflammation-draining LNs. Although no differences in the proportions of B220+, TCRβ+, CD4+ and CD8+ cells were observed when comparing WT with K14-VEGFC inflamed LNs (Figure 2K), inflammation-draining LNs of K14-VEGFC mice had significantly fewer B220+, TCRβ+, CD4+ and CD8+ cells, indicative of a reduced inflammatory response (Figure 2L). Since migratory activated DCs from sites of inflammation present antigens to T cells within draining LNs, the activity of T cells within the draining node should reflect the frequency and activation status of intra-nodal DCs. We therefore isolated CD8+ cells from untreated and inflamed mice and examined IFN-γ production following *in vitro* stimulation with PMA/ionomycin. In agreement with our observation that draining LNs of K14-VEGFC mice had reduced numbers of DCs exhibiting a more immature phenotype, inguinal LNs of K14-VEGFC mice contained significantly fewer IFN-γ-producing CD8+ cells. These differences were not observed in untreated mice or non-skin-draining (mesenteric) LNs (Figure 2M-2N).

### VEGF-C does not directly modulate the phenotype of dendritic cells

We next tested whether DCs express VEGFR3, the receptor for VEGF-C. CD11c+BMDCs significantly upregulated VEGFR3, and to a lesser extent VEGFR2 and VEGFR1, in response to IFN-γ or TPA stimulation (Figure 3A). However, VEGFC was not chemotactic for control or IFN-γ-stimulated BMDCs, whereas CCL21 promoted chemotactic migration (Figure 3B). Expression of CD40 (Figure 3C), CD80 (Figure 3D), and CD86 (Figure 3E) was not significantly altered on control, IFN-γ- or TPA-stimulated BMDCs upon addition of VEGF-C (500 ng/mL). Similarly, recombinant VEGF-C had no effect on the expression of the MHCII invariant chain I-A/I-E (Figure 3F). The ability of VEGF-C to modulate the expression of pro- and anti-inflammatory cytokines (IL-6, IL-10, TNFα) (Figure 3G, 3H and 3I), the chemokine CCL2 (Figure 3J) and the expression of the chemokine receptor CCR7 (Figure 3K) was also analysed using realtime PCR. In unstimulated and IFN-γ- or TPA-stimulated cells, VEGFC had very minor to no effects on the expression of these genes.

### Lymphatic endothelial cell conditioned media induce immunosuppressive dendritic cells

**Figure 4:**
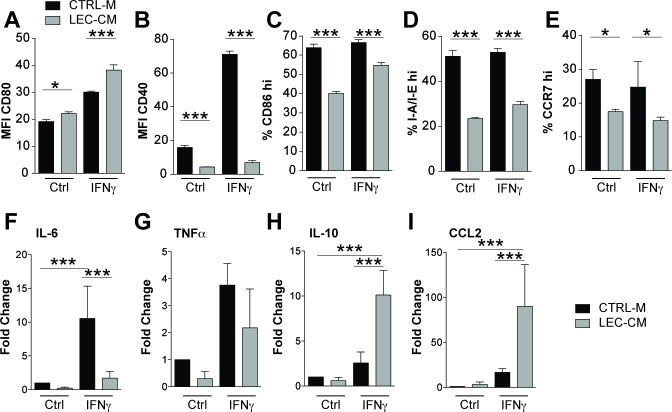
Lymphatic endothelial cell conditioned media induce an immunosuppressive phenotype in bone marrow derived CD11c+ cells Bone marrow derived CD11c+ cells were treated with control media (CTRL-M) or lymphatic endothelial cell-conditioned media (LEC-CM) for 24 hours with or without IFN-γ stimulation (100 ng/mL). Flow cytometric analysis of their expression of CD80 (**A**), CD40 (**B**), I-A/I-E (**C**), CD86 (**D**) and CCR7 (**E**) was performed with either the median fluorescence intensity (MFI) or percentage of cells expressing high marker levels shown. Data shown are representative of three independent biological replicates. mRNA isolated from likewise treated cells was analysed using RT-PCR for the expression of IL-6 (**F**), TNFα (**G**), IL-10 (**H**) and CCL2 (**I**) Data are shown as the mean fold change relative to control treated cells (normalized to 1) of three independent biological replicates. Statistical significance was assessed using the Two-way (A-E) and One-Way (F-I) ANOVA with Bonferroni post-tests. *p < 0.05, **p < 0.01, ***p < 0.001.

As VEGF-C had no direct effect on DC maturation, we investigated if LECs might produce factors that modulate the phenotype of DCs. BMDCs were treated with conditioned media collected from LECs (LEC-CM) and cultured with or without IFN-γ. Using flow cytometry, these cells were assessed for expression of CD80, CD40, CD86, I-A/I-E and CCR7 (Figure 4A-4E). Treatment of BMDCs with LEC-CM significantly downregulated expression of co-stimulatory CD40 and CD86, reduced the expression of I-A/I-E and also significantly reduced CCR7 expression. We then analyzed the inflammatory cytokine profiles of these DCs using quantitative RT-PCR and found that treatment with LEC-CM also significantly reduced the expression of pro-inflammatory IL-6 (Figure 4F) when stimulated with IFN-γ. TNF-α was also reduced under control and IFN-γ stimulation, however, this was not statistically significant (Figure 4G). Furthermore, LEC-CM significantly upregulated the anti-inflammatory cytokine IL-10 (Figure 4H) and the chemokine CCL2 (Figure 4I). Collectively, these data reveal that LECs produce a factor, or factors, that potently induce an immature, less inflammatory phenotype in DCs, characterized by a potentially reduced capacity for antigen presentation and an anti-inflammatory cytokine production profile. Given the observed elevated TGF-β levels in inflamed skin of VEGF-C mice (Figure 1I) and the ability of LECs to produce TGF-β (Figure 1J), we investigated if TGF-β could induce the production of DCs with a less mature phenotype. Addition of TGF-β during DC maturation assays had no effect on the expression of I-A/I-E or CD40 (Supplementary Figure 4). Furthermore, addition of TGF-β signaling inhibitors (LY-364947 and SB-431542) failed to block the potent maturation inhibitory effect of LEC-CM, indicating that TGF-β does not play a major role in the LEC-induced inhibition of DC maturation (Supplementary Figure 4).

### Blockade of prostaglandin synthesis partially reverts LEC-CM effects on dendritic cell function

**Figure 5:**
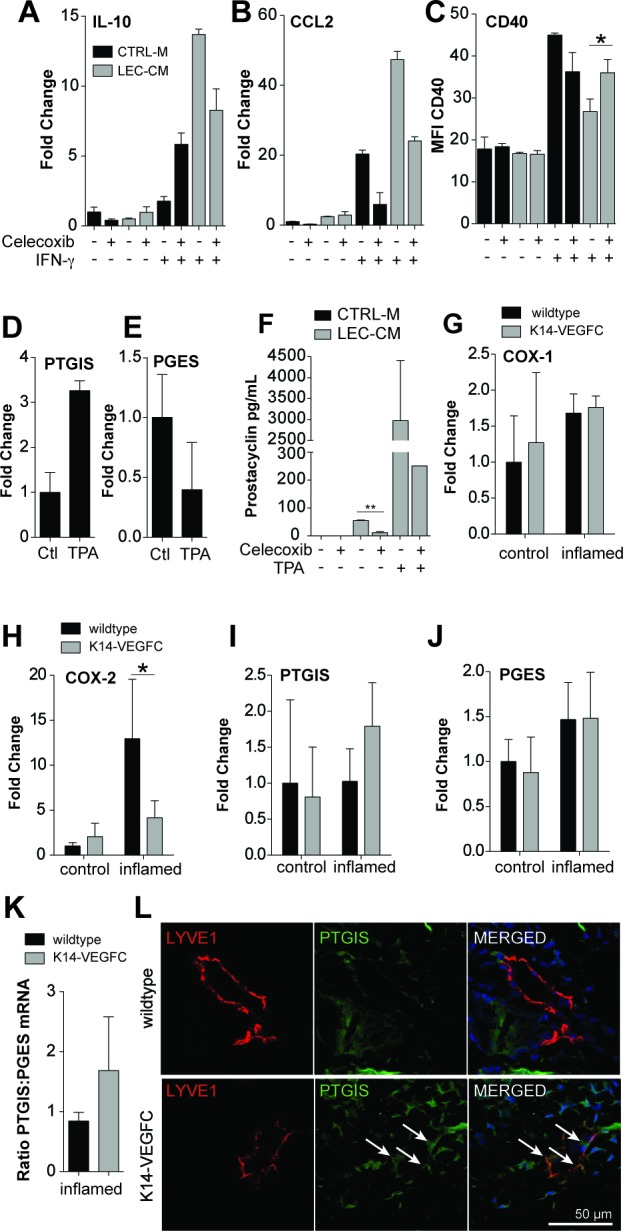
Blockade of prostaglandin synthesis partially reverts immunosupressive effects of LEC conditioned media Bone marrow derived CD11c+ cells were treated for 24 hours with either control media (CTRL-M) or lymphatic endothelial cell-conditioned media (LEC-CM) that was generated ± Celecoxib (20 μM). BMDCs were stimulated with ± IFN-γ (100 ng/mL) as shown. mRNAs from treated cells were then analysed using RT-PCR for the expression of IL-10 (**A**) and CCL2 (**B**) Data are shown as the fold change relative to control treated cells and are representative of three independent biological replicates. Likewise treated cells were also analysed using flow cytometry for their expression of CD40 (**C**) Data are shown as the median fluorescence intensity and are representative of two biological replicates. LECs were stimulated with TPA (10 ng/mL) for 6 hours and the relative expression of prostacyclin synthase (PTGIS) (**D**) and prostaglandin E synthase (PGES) (**E**) compared to *Rplp0* was assessed using real-time PCR. Data are shown as the fold change relative to control treated cells (normalized to 1) and are representative of three independent biological replicates. Prostacyclin levels in cell culture supernatants were assessed *via* ELISA. LECs were cultured for 48 hours with or without celecoxib (20 μM) and TPA (10 ng/mL) (**F**) RT-PCR of mRNA extracted from the skin of wildtype and K14-VEGFC, control (*n* = 3) and inflamed (*n* = 3-4) mice was used to assess the relative expression of COX-1 (**G**), COX-2 (**H**), PTGIS (**I**) and PGES (**J**) when compared to *Rplp0.* Data are shown as the fold change ± SD relative to control-treated wildtype mice (normalized to 1). The ratio of the relative expression of PTGIS:PGES is shown in (**K**)Representative confocal images of inflamed back skin of wildtype and K14-VEGFC mice, stained for LYVE-1 (red) and PTGIS (green), scale bar 50 μm (**L**) We consistently observed more PTGIS staining in K14-VEGFC skin, both in lymphatic endothelial cells (arrows) and non-endothelial cells. Note that lymphatic vessels of similar size were chosen for better comparability of the staining pattern. Student's t-test was used to assess statistical significance in A-C, F and K. Two-way ANOVA with Bonferroni's post-test was used in G-J. *p < 0.05.

Prostaglandins are potent modulators of inflammation. We next investigated if prostaglandins might contribute to the immunosuppressive effects of LEC-CM on DCs. BMDCs were cultured with CM generated from LECs treated or not with the COX-2 inhibitor Celecoxib and were analysed using RT-PCR and flow cytometry for expression of key cytokines, chemokines and surface markers. Blockade of COX-2 reduced LEC-CM-mediated upregulation of IL-10 expression in BMDCs by approximately 40% (Figure 5A) and also reduced LEC-CM-induced upregulation of CCL2 expression by approximately 50% (Figure 5B). Celecoxib treatment of LECs also partially reversed LEC-CM-mediated downregulation of CD40 expression on the surface of BMDCs (Figure 5C), potentially restoring effective antigen presentation function of these cells.

The generation of specific prostaglandins is dependent upon the availability of their respective synthases. We analyzed in cultured LECs the expression of two downstream prostaglandin synthases, prostacyclin (prostaglandin I2, PGI_2_) synthase (PTGIS) and prostaglandin E synthase (PGES). LECs increased the expression of PTGIS approximately 3-fold (Figure 5D) upon treatment with TPA, and reduced the expression of PGES to approximately 40% of that expressed by control cells (Figure 5E). In line with this, cultured LECs were found to produce prostacyclin (assessed by ELISA of cell culture supernatants), and upregulated its production upon stimulation with TPA (Figure 5F). Importantly, blockade of prostaglandin synthesis using Celecoxib significantly reduced prostacyclin levels in LEC-CM (Figure 5F).

We next investigated the levels of prostaglandin synthases in the skin of mice. The relative expression levels of COX-1 were largely unchanged following inflammation (Figure 5G), whereas COX-2 was strongly upregulated in the inflamed skin of WT mice (Figure 5H). Strikingly, a similar upregulation of COX-2 was not observed in the inflamed skin of K14-VEGFC mice with expression levels similar to those observed in uninflamed skin (Figure 5H). PTGIS expression was not increased in inflamed WT mice but was upregulated in the inflamed skin of K14-VEGFC mice (Figure 5I). PGES expression was comparably expressed in WT and K14-VEGFC mice (Figure 5J). In inflamed skin, K14-VEGFC had an approximately two fold higher ratio of PTGIS to PGES than WT mice (Figure 5K). In accordance with these findings, the inflamed skin of K14-VEGFC mice showed lymphatic PTGIS staining that was largely absent from the skin of inflamed wildtype controls (Figure 5L). Thus, an increase in the levels of anti-inflammatory (PTGIS) *versus* pro-inflammatory (PGES) prostaglandin synthases may contribute to the immune-inhibitory microenvironment in the lymphatic rich skin of VEGF-C transgenic mice.

## DISCUSSION

Topical TPA application to the skin induces cutaneous inflammation characterized by increased vascular permeability, swelling and edema, inducing a significant inflammatory cell infiltration within the dermis [35–37]. We have previously reported that transgenic expression of VEGF-C in the skin, resulting in an expanded dermal lymphatic network, limits acute skin inflammation and reduces dermal edema formation in response to challenge by oxazolone and UV-B [5]. Interestingly, the application of oxazolone to K14-VEGFC mice revealed a significant increase in CD11b+ cells when compared to WT controls [5]. In the current study, we have more comprehensively characterized the inflammatory infiltrate in response to TPA, and identified a new mechanism how LECs contribute to the attenuation of inflammation.

DCs, the major antigen presenting leukocytes, play crucial roles in inflammation and immunity. Importantly, we found that inflamed skin of K14-VEGFC mice contains significantly higher proportions of CD11c+CD11b+ cells than WT mice. Although increased in number, these cells were less mature, with significantly reduced levels of MHCII, co-stimulatory molecules, and CCR7, suggesting a naïve, tolerogenic phenotype. A defect in DC migration to the draining LNs could result in their accumulation in the skin. Indeed, we found that the ability of DCs to traffic to draining LNs was severely compromised in inflamed skin of K14-VEGFC mice, most likely due to their reduced expression of the key migratory chemokine receptor CCR7 [26, 38].

Clearly, impairment of DC trafficking can severely impact upon the successful generation of active immune responses. As a result, the inflamed skin-draining LNs of K14-VEGFC mice were significantly reduced in their weight, size and cellularity when compared with inflamed WT LNs. Lymph node resident CD11c+CD11b+ cells exhibited reduced antigen presentation capabilities with I-A/I-E and CD40 expressed at lower levels when compared to those in inflamed WT LNs. Importantly, the ability of CD8+ cells in inflamed K14-VEGFC LNs to produce IFN-γ upon *in vitro* stimulation was significantly reduced when compared to those isolated from inflamed WT mice, indicating that skin-derived DCs were not able to activate T-cells efficiently.

Concomitant with a less activated DC phenotype, we also observed an increase in CD4+Foxp3+ Tregs in the inflamed skin of K14-VEGFC mice when compared to WT controls. This is in line with the reported increase of infiltrating Tregs in VEGF-C overexpressing tumour models [19]. Tregs play a critical role in the resolution of inflammation, suppressing both DC and effector T-cell functions (reviewed by [39]). A key immune-regulatory cytokine is TGF-βl, which is produced by multiple cell types including Tregs and DCs, and at the same time regulates the activity of both these cell types (reviewed in [40]). For example, TGF-β1 skews DCs towards a tolerogenic phenotype [29, 41], while also modulating the generation and regulatory effects of Tregs [31, 42, 43]. Thus, the observed increased levels of TGF-β1 likely contribute to the immune-inhibitory microenvironment in the inflamed skin of K14-VEGFC mice. Importantly, our data further indicate that LECs are a prominent source of TGF-βl. Given the largely expanded lymphatic network that we observed in K14-VEGFC mice under inflammatory conditions, it is conceivable that lymphatic-derived TGF-βl contributes to immune inhibition in the dermis. However, our data suggest that TGF-βl is not the main factor responsible for the immature DC phenotype observed in the skin of K14-VEGF-C mice.

Inhibition of DC maturation has been reported for the closely related vascular endothelial growth factor-A (VEGF-A) *in vitro* and *in vivo* [44, 45]. Given that in the presence of elevated VEGF-C DCs were less activated, we first hypothesized that, similar to VEGF-A, VEGF-C might also exert tolerogenic effects on DCs. However, although we found upregulation of VEGFR-3 in DCs, we could not find any direct effect of VEGF-C on those cells *in vitro*.

Alternatively, the expanded lymphatic endothelium may modulate the maturation of DCs in K14-VEGFC mice. Previously, inflamed LECs were found to downregulate DC expression of costimulatory CD86 *via* an ICAM-1/Mac-1 dependent adhesive interaction that in turn suppressed the ability of DCs to activate T-cells [46]. Our data suggests that, in addition to TGF-β1, LECs may produce other factors that modulate DC maturity. Notably, when culturing BMDCs in LEC conditioned media, they assumed an immature phenotype, reminiscent of the DC phenotype in the inflamed skin of K14-VEGFC mice. To our knowledge, this is the first time that LEC-derived factors have been found to directly impact upon DC maturity. Furthermore, LEC-CM also significantly altered DC cytokine profiles, with reduced expression of pro-inflammatory IL-6 and TNF-α following stimulation with IFN-γ and simultaneous upregulation of anti-inflammatory IL-10. Notably, secretion of IL-10 by DCs drives the development of TGF-β- and IL-10-secreting Tregs, providing a positive feedback loop for its induction [47, 48]. LEC-modulated DCs with an increased expression of IL-10 would thus significantly contribute to an immune-inhibitory microenvironment in K14-VEGFC mice. In addition to IL-10, LEC-CM also strongly primed DC for the expression of CCL2. CCL2 together with its receptor CCR2 is involved in the migration of various immune cells, such as monocytes and macrophages [49], effector T-cells [50] but also Tregs [51]. This may be an additional mechanism whereby increased numbers of Tregs are recruited to the inflamed dermis in K14-VEGFC mice. Furthermore, exposure of splenic T-cells to recombinant CCL2 decreased their ability to produce IFN-γ [52] suggesting CCL2 may also inhibit effector T-cells directly.

One potential class of molecules with powerful DC modulatory capabilities are prostaglandins [53, 54]. Blockade of LEC prostaglandin synthesis using celecoxib reduced the anti-inflammatory effects of LEC-CM by reducing DC IL-10 and CCL2 expression, and partially restoring CD40 expression. To further elucidate the mechanisms underlying prostaglandin-mediated alterations in DC maturation, we analysed the relative expression of two of the key prostaglandin synthase enzymes, prostacyclin synthase (PTGIS) and prostaglandin E synthase (PGES). *In vitro*, under inflammatory conditions, LECs upregulated *PTGIS* while *PGES* was downregulated. This finding was supported with inflamed LECs significantly upregulating prostacyclin production. Prostacyclin limited inflammation in *in vivo* models of viral infection and allergic responses [55–57]. More recently, it has been shown that prostacyclin analogues decreased proinflammatory cytokine secretion (including IL-6 and IL-1β), upregulated IL-10 production and decreased co-stimulatory and MHCII expression on DC [53], similar to the effects observed here. Conversely, PGE_2_ is largely considered pro-inflammatory, with PGES^−/−^ mice exhibiting decreased dermal inflammatory responses and reduced disease severity in a model of rheumatoid arthritis [58, 59]. With regards to DC function, PGE_2_ enhanced co-stimulatory molecule and MHCII expression, induced proinflammatory cytokine secretion [54], and has been shown to augment antigen-specific CD4+ and CD8+ T-cell proliferation [60]. Thus, we believe that a shift towards the generation of anti-inflammatory prostacyclin, together with a decrease in pro-inflammatory PGE_2_plays a major role in the attenuation of dermal inflammation by the lymphatic endothelium.

*In vivo,* COX-2 was surprisingly not upregulated in the inflamed skin of K14-VEGFC mice compared to WT mice. On the other hand, these mice expressed higher levels of PTGIS, while PGES expression was equivalent between the two genotypes. Functional coupling of PGES to COX-2 in preference to COX-1 [61–64] and preferential selectivity of PTGIS to COX-1 [65–68] may add an additional level of regulation to prostaglandin synthesis. Low levels of COX-2 may reduce PGES activity, while a concomitant relative increase in PTGIS activity would allow anti-inflammatory prostacyclin synthesis to dominate. Future studies using a lymphatic specific knockout of PGES or PTGIS would help to dissect the contribution of this mechanism to the regulatory activity of LEC with regard to DC phenotype and inflammation.

**Figure 6:**
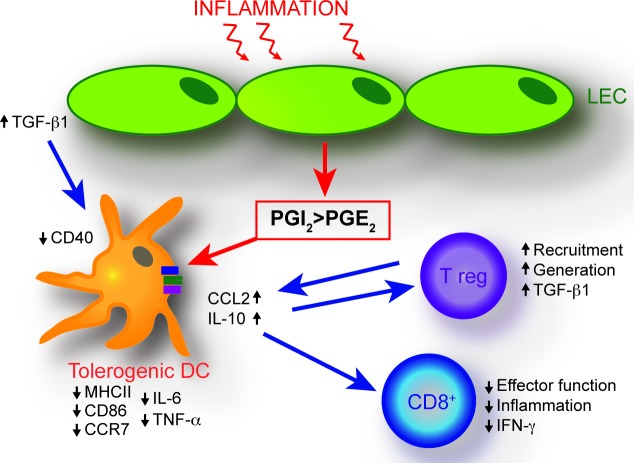
Schematic of proposed mechanisms of LEC-mediated dendritic cell modulation The VEGF-C-mediated expansion of the inflammatory lymphatic vessel network results in an immune-inhibitory dermal microenvironment. An imbalance in LEC synthesis of prostaglandins, namely a shift towards increased synthesis of the inflammation limiting prostacyclin, concomitant with decreased synthesis of the pro-inflammatory PGE_2_, represents a key mechanism resulting in the generation of immature DCs with an anti-inflammatory cytokine profile. These effects are compounded by increased LEC-derived TGF-β1 production. In addition, the dermal cellular milieu exhibits elevated numbers of Tregs. Decreased DC maturation is implicated in the generation of CD8+ T cells with a reduced ability to produce pro-inflammatory IFN-γ.

Collectively, this study highlights that in addition to enhancing lymphatic drainage by increasing the expansion of lymphatic vessels [5, 69, 70], VEGF-C-induced LECs also possess immune modulating properties that act to reduce inflammation. We have identified LEC-derived prostaglandins as a negative modulator of DC maturation and anti-inflammatory cytokine production in the skin. This effect may be compounded by increased LEC-derived TGF-β1 production (Figure 6). As a result, DC maturation and CD8+ T-cell activation are inhibited under inflammatory conditions in VEGF-C overexpressing mice, whereas Tregs are elevated. LEC-mediated immunological changes therefore may further impact positively upon the resolution of inflammation. Together, these findings reveal an unanticipated role of the lymphatic endothelium in dampening inflammation *via* distinct molecular and cellular mechanisms.

## MATERIALS AND METHODS

### Mouse model of TPA-induced inflammation

Chronic dorsal skin inflammation was induced in 6-8 week old hemizygous K14-VEGFC [20] and littermate WT mice with TPA application (5 μg dissolved in acetone 1x/week for 5 weeks). Experimentation was undertaken in accordance with protocols approved by the Kantonales Veterinäramt Zürich. Tissue for analysis was harvested two days after the final TPA treatment. Protein was isolated from back skin of known weight as described [5]. VEGF-C and TGF-β1 protein was quantified using VEGF-C and TGF-β1 ELISA Kits (R&D Systems), and was normalised to tissue weight and total protein amount within the lysate. Immunofluorescence stainings were performed using standard techniques as described in the Supplementary Methods. Stained sections were examined on an Axioscope Mot Plus microscope (Carl Zeiss) equipped with an Axiocam MRc camera (Carl Zeiss). Images were acquired using Axio-Vision software Version 4.7.1 (Carl Zeiss). ImageJ was used for image analysis. To quantify immune cell infiltration and the area covered by lymphatic vessels in the skin of mice, ten images/skin section/mouse at a 20x magnification were taken. Foxp3+CD4+ cells were quantified by manually counting double positive cells in skin sections from three control and four inflamed mice for each genotype.

### Flow cytometric analysis of tissue samples

Single cell suspensions were prepared from tissues and stained as described in the Supplementary Methods. Antibodies used are listed in Supplementary Methods Table 2. Flow cytometry was performed using a FACS Canto Flow Cytometer (BD) and data was analysed using FlowJo software. The expression levels of activation markers are shown as the median fluorescence intensity (MFI).

### *In vivo* dendritic cell migration

Inflamed back skin of mice was painted with fluorescein isothiocyanate (FITC - 0.5%, Thermo Scientific) dissolved in acetone/dibutyl phthalate (1:1 vol/vol - Sigma) as described [71, 72]. Eighteen hours later, inguinal and axillary lymph nodes were harvested, cellularity determined and the proportions of FITC+CD11c+CD11b+ cells were quantified (Schematic shown in Figure 2H) using flow cytometry.

### Quantitative RT-PCR

RNA was isolated as described in the Supplementary Methods. Gene expression was investigated by quantitative RT-PCR using FastStart Universal SYBR Green Master Mix (Roche), the 7900HT Fast Real-time PCR system (Life Technologies), and quantified using the 2^−ΔΔCt^ method. Primers (Microsynth) are shown in Supplementary Methods Table 3. All data were normalized to the expression of the reference gene Rplp0.

### Generation of LEC-conditioned media

Human dermal lymphatic endothelial cells [73] were cultured on collagen coated (50 μg/mL) tissue culture plates in EBM (Lonza) + 20% FBS (Life Technologies), 1x penicillin/streptomycin (Life Technologies), 2 mM L-glutamine (Life Technologies), 25 μg/ml cAMP (Sigma-Aldrich), and 10 μg/ml hydrocortisone (Sigma-Aldrich), until approximately 80% confluent. Cells were washed twice with PBS and cultured for 72 hours in 1% FBS EBM ± 20 μM Celecoxib (Sigma). Harvested cell culture supernatants were centrifuged and supernatants stored at −80°C.

### *In vitro* dendritic cell assays

CD11c+ cells (BMDCs) were generated from bone marrow as described in the Supplementary Methods. BMDCs were plated in triplicate per treatment group into U-bottom 96 well plates at 2×10^5^/well and stimulated for 24 hours with IFN-γ (100 ng/mL) or TPA (20 ng/mL). Recombinant human VEGF-C (R&D) was used at 500 ng/mL. When assessing the effects of LEC-CM, BMDCs were cultured in 96 well plate wells in 10 μL complete media, 10 μL 1% FBS EBM and 50 μL LEC-CM for FACS analysis. For RNA extraction, 5×10^6^ BMDCs were plated into 24 well plates and incubated with complete EBM:LEC-CM (1:1). For transwell migration assays, BMDCs were incubated ± IFN-γ (100 ng/mL). The following day, 2×10^4^ cells were seeded onto 24-well transwell inserts (pore size 5 μm, Corning), in triplicates. The medium in the bottom chamber consisted of 1% FBS EBM and 500 ng/mL VEGF-C (R&D) or 100 ng/mL CCL21 (R&D). Cells were incubated for 4 hours, the lower chamber medium was removed and the cell number was quantified using flow cytometry and Accucheck counting beads (Invitrogen).

### Statistical analyses

Histological parameters were measured in a blinded fashion. All data are expressed as the mean ± SD as stated in the figure legends. Statistical significance was assessed using the two-tailed unpaired Student's *t* test or Two-way ANOVA with Bonferroni's multiple comparison test as stated in the figure legends. A value of *p* < 0.05 was taken to be statistically significant.

## SUPPLEMENTARY MATERIAL FIGURES AND TABLES


