# Supplementary material for: Lymphatic endothelial cells attenuate inflammation via suppression of dendritic cell maturation
Source: Oncotarget. 2016 Jun 5;7(26):39421–35. doi: 10.18632/oncotarget.9820 (PMC5129942; doi:10.18632/oncotarget.9820)
Supplement: Supplementary file 1 [file oncotarget-07-39421-s001.pdf]

Lymphatic endothelial cells attenuate inflammation *via* suppression of dendritic cell maturation

Supplementary Material

Immunofluorescent Histology

For immunofluorescent histochemistry, freshly harvested tissues were embedded in optimal cutting temperature (O.C.T.) compound (Tissue-Tek®, Sakura) and frozen in liquid nitrogen before being stored at -80°C. For immunofluorescent staining, 8 µm cryo-sections of skin and inguinal lymph nodes were cut. All sections were placed on Superfrost® Plus glass slides (Thermo Scientific) and air dried before being stored at -80°C. For immunofluorescent staining, skin and lymph node sections were fixed for 4 minutes in -20°C acetone and then subsequently rehydrated with 4°C 80% methanol, phosphate buffered saline (PBS), and PBS with 12% bovine serum albumin (BSA) prior to staining with primary antibodies. Primary antibodies (Supplementary Table 1) were diluted in antibody diluent (Zytomed) and applied to the sections. Sections were stained with the relevant secondary antibodies (Life Technologies) and Hoechst 33342 nuclear dye (10 µg/ml in PBS, Life Technologies) in PBS. Finally, slides were washed then mounted with Mowiol (EMD Biosciences). Stained sections were examined on an Axioscope Mot Plus microscope (Carl Zeiss) equipped with an Axiocam MRc camera (Carl Zeiss). Images were acquired using Axio-Vision software Version 4.7.1 (Carl Zeiss). To quantify immune cell infiltration and the area covered by cutaneous lymphatic vessels in the skin of mice, ten images/skin section/mouse at a 20x magnification were taken. ImageJ was used for image analysis.

Whole Mount Immunostaining

Whole mount immunostainings were performed as previously described [1]. Briefly, ears from 9 week old wildtype and K14-VEGFC mice were removed, split in half and the outer part of the ear was used for analyses. Lymphatic vessel junctions were visualized using a LYVE-1 antibody (AngioBio) together with a VE-Cadherin antibody (R&D) (Supplementary Table 1). Ears were fixed for 2 hours in 4% PFA at 4°C, then washed thoroughly with PBST and blocked for 2 hours at room temperature with immunomix (5% donkey serum, 0.1% Triton-X, 1% BSA, 0.05% NaN_3). Ears were then incubated with the primary antibodies diluted in immunomix overnight at 4°C, washed

thoroughly with PBST and then stained with the relevant secondary antibodies. Following staining, samples were flat-mounted on glass slides using Mowiol (Calbiochem) and images were obtained using a Zeiss 780 confocal microscope. The acquired images were processed using ImageJ and Adobe Photoshop CS5 (Adobe Systems). Representative images are shown from that observed in three wildtype and three K14-VEGFC mice.

Single cell suspension preparation for Flow cytometry analysis

Single cell suspensions of LNs, spleen and thymus were prepared by manual disruption through 40 μm cell strainers. Erythrocytes in blood and spleen samples were lysed using Pharma Lyse™ (BD). Skin was digested in a collagenase IV solution (10 mg/ml Collagenase IV (Life Technologies), 20 μg/ml DNase (Life Technologies), 2.25 μM $CaCl_2$ in PBS), at 37°C for 30 minutes and the tissue then filtered sequentially through 70 μm and 40 μm cell strainers.

Intracellular staining for Flow cytometry analysis

For intracellular IFN-γ staining, single cell suspensions from inguinal and mesenteric LNs were incubated with PMA (10 ng/ml, Sigma), ionomycin (500 ng/ml, Sigma) and BD GolgiPlug for 4 hours. Cells were then stained using the BD Cytofix/Cytoperm™ kit.

Generation of BMDCs

Bone marrow was flushed from the tibia and femur of wildtype mice and cultured at a density of 5-10x10^5 cells/mL in complete RPMI media containing 20 ng/mL GM-CSF (R&D) in non-tissue culture treated 100 mm diameter dishes for a total of six days. After three days culture, GM-CSF containing media was refreshed. On day six, the non-adherent cells were harvested and the CD11c+ cells purified using standard MACS cell sorting techniques (Miltenyi Biotech). CD11c+ purity was analysed by flow cytometry and was always greater than 95%.

RNA isolation

Total cellular RNA was isolated from mouse back skin using a TissueLyser and the PureLink™ RNA Mini Kit (Ambion) or extracted from *in vitro* treated DCs (24 hour treatment) and LECs (6 hour treatment) using a Nucleospin RNA kit (Macherey-Nagel). RNA was transcribed to cDNA using the High-Capacity cDNA Reverse Transcription Kit (Life Technologies).

***In vivo* Lymphatic Clearance Assay**

Clearance from back skin was assessed by measuring the disappearance over time of a pegylated near-infrared dye (P20D800) as previously described [2]. In brief, 3 μL of 3 μM P20D800 tracer were injected intradermally into the back skin and imaging was performed using an IVIS spectrum (Caliper Life Sciences) at 0, 1, 2, 3, 4 and 6 hours after injection (λ_{ex}: 745 nm, λ_{em}: 800 nm, exposure time 4 s). Fluorescence intensities were measured as fluorescence counts, adjusted to baseline values and normalized to the values directly after injection. Data were then fit to a one-phase exponential decay model using GraphPad Prism to determine the half-life (Half Life = ln 2 / K , expressed in hours) as a measure of lymphatic clearance.

1. Karaman S, *et al.* Blockade of VEGF-C and VEGF-D modulates adipose tissue inflammation and improves metabolic parameters under high-fat diet. *Mol Metab.* 2015; 93-105: 4.

2. Karaman S, Buschle D, Luciani P, Leroux JC, Detmar M, Proulx ST. Decline of lymphatic vessel density and function in murine skin during aging. *Angiogenesis* 2015; 489-498: 18.

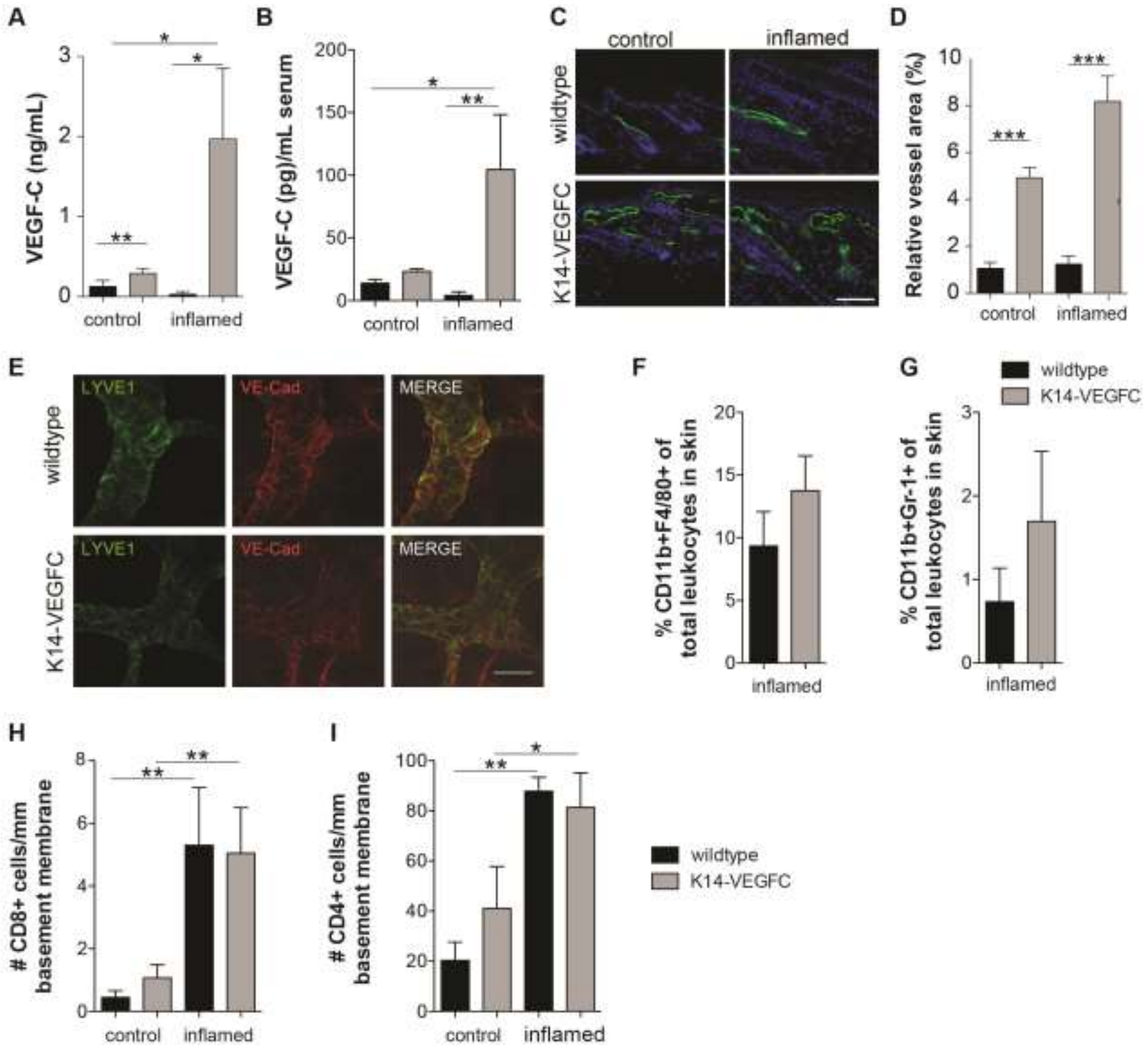

Supplementary Figure 1: TPA-inflammation induces VEGF-C expression and lymphangiogenesis
VEGF-C protein levels (per mg total protein) were quantified in the skin and sera of untreated (n=4 per genotype) and inflamed (n=4 per genotype) K14-VEGFC transgenic and littermate wildtype control mice using ELISA (**A** and **B**). Data are presented as mean ± SD. Representative images of the expanded lymphatic vasculature observed upon inflammation are shown in **C** (scale bar: 100 μm). Relative area occupied by lymphatic vessels is quantified in (**D**), untreated n=3 per group, inflamed n=5 per group. Representative confocal images of ear tissue whole mount immunostainings are shown in **E**. LYVE-1 (green), VE-cadherin (red) and a merged image are shown for wildtype and K14-VEGFC mice, scale bar: 50 μm. Flow cytometry was used to determine the proportions of CD11b+F4/80+ (**F**) and CD11b+Gr-1+ (**G**) cells in the skin of inflamed WT (n=4) and K14-VEGFC (n=4) mice. Skin sections from untreated (n=3) and inflamed (n=4) WT and K14-VEGFC mice were stained for CD8 and CD4 and quantified (**H** and **I**). Although CD4+ and CD8+ cells significantly increased upon inflammation, no differences were observed when comparing K14-VEGFC to WT mice. Data are presented as mean ± SD. *p<0.05, **p<0.01 ***p<0.001.

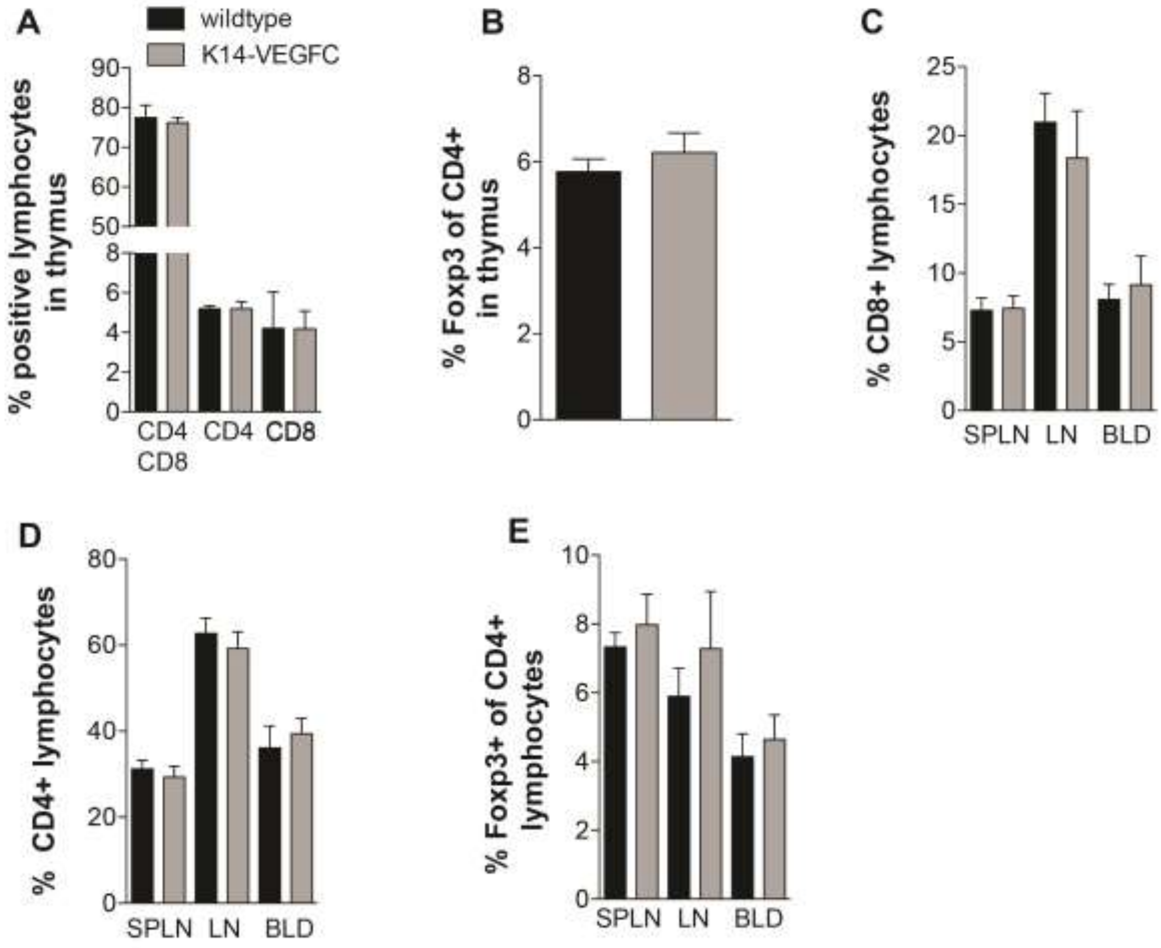

Supplementary Figure 2

Flow cytometry was used to quantify the proportion of CD4+, CD8+, and CD4+CD8+ cells in the thymus (**A**). The proportion of CD4+ cells that were Foxp3+ in the thymus was also quantified (**B**). Flow cytometry was also used to quantify the proportions of CD8+ (**C**), CD4+ (**D**) and Foxp3+ cells of CD4+ cells (**E**) in the spleen, inguinal LNs and blood. No significant differences were observed between WT (n=4) and K14-VEGFC (n= 5).

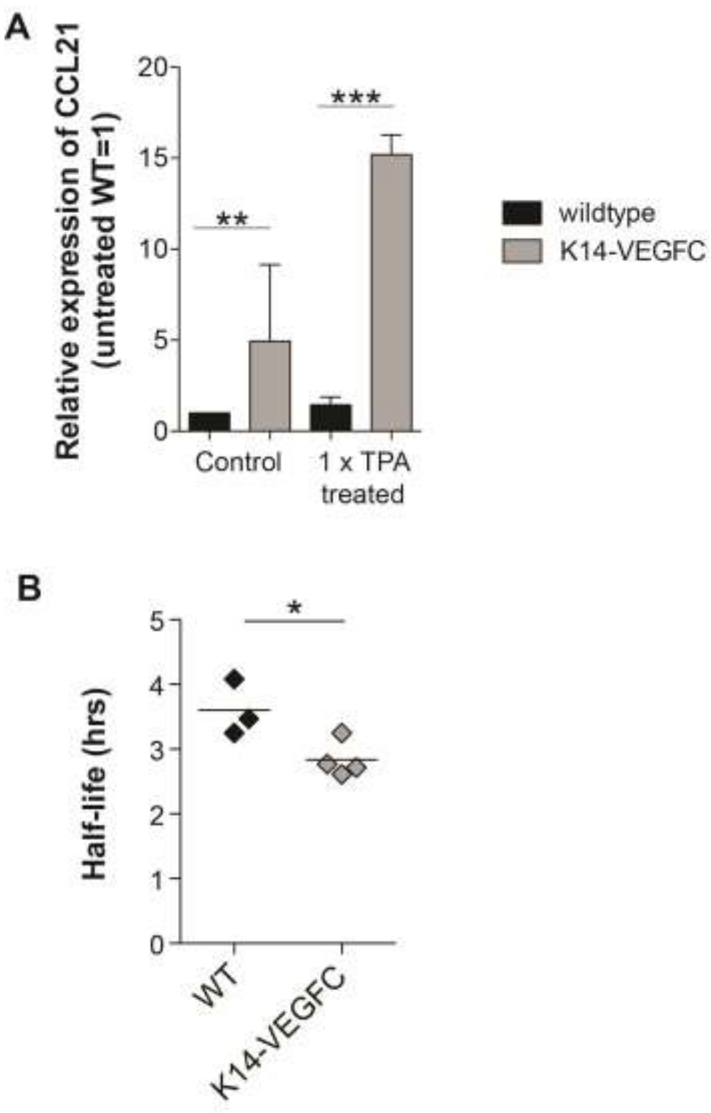

Supplementary Figure 3

The relative expression of CCL21 mRNA when compared to Rplp0 was measured by RT-PCR in the skin of untreated (n=3) and 1 x TPA treated (n=4) mice (**A**). The lymphatic specific tracer P20D800 was injected intradermally into the inflamed dorsal skin of wildtype and K14-VEGFC mice. Half-life of the tracer was calculated based on the amount that remained in the skin over time (**B**). Data are presented as mean ± SD. Statistical significance was assessed using the Student's t-test.*p<0.05, **p<0.01, ***p<0.001.

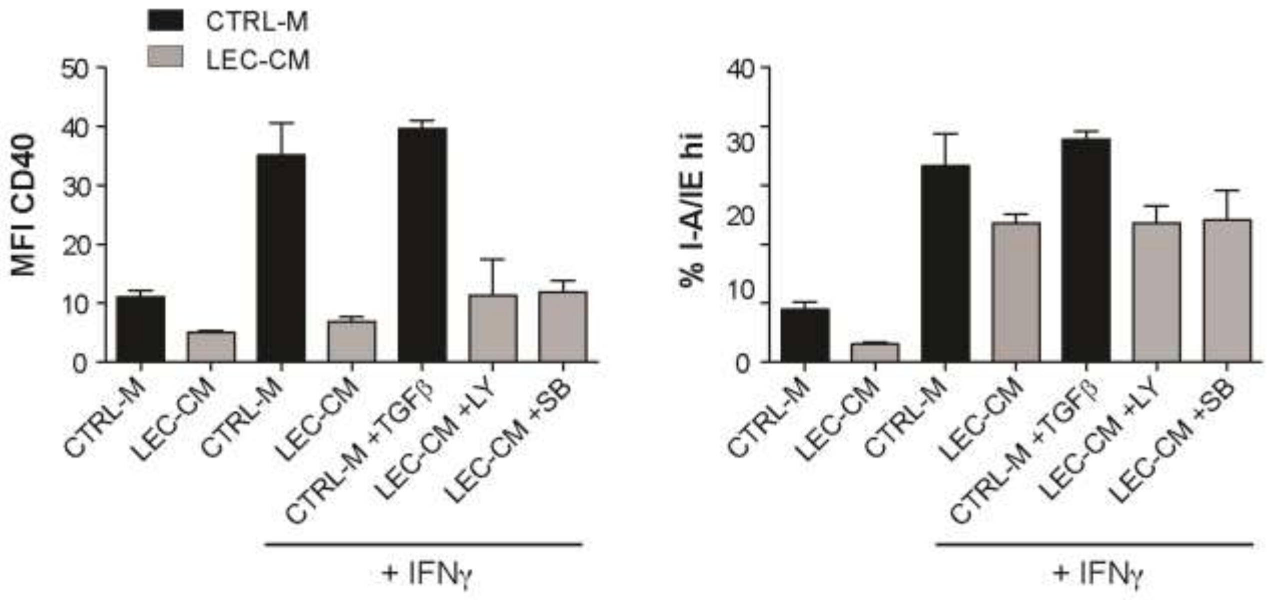

Supplementary Figure 4: TGF-β does not account for the observed effects of LEC-CM on bone marrow derived dendritic cells.

BMDCs were treated with LEC-conditioned media containing IFN-γ (100 ng/mL) and the small molecule inhibitors LY-364947 and SB-431542 (LY and SB respectively, both at 1 μM) and were cultured for 24 hours. Expression of I-A/I-E and CD40 on CD11c+Cd11b+ cells was assessed via flow cytometry. TGF-β (100 ng/mL) alone had no effect on the expression of either marker. The inhibitors also failed to reverse the inhibitory effects of LEC-CM on DC maturation.

Supplementary Table 1: Antibodies used for immunofluorescent histology staining

Antigen	Clone	Dilution	Supplier
**B220**	RA3-6B2	1:200	Becton Dickinson
**CD4**	H129.19	1:100	Becton Dickinson
**CD8a**	53-6.7	1:100	Becton Dickinson
**Foxp3**	FJK-16S	1:100	eBiosciences
**LYVE-1**	Polyclonal	1:600	AngioBio
**VE-Cadherin**	Polyclonal	1:50	R&D
**PTGIS**	Polyclonal	1:250	Abcam

Supplementary Table 2: Antibodies used for Flow cytometry

Antigen	Clone	Dilution	Supplier
**B220**	Ra3-6B2	1:200	Becton Dickinson
**CD3**	17A2	1:200	Biolegend
**CD4**	GK1.5	1:200	BioLegend
**CD8a**	53-6.7	1:200	Biolegend
**CD11b**	M1/70	1:200	Becton Dickinson
**CD11c**	N418	1:200	BioLegend
**CD40**	3/23	1:200	Becton Dickinson
**CD80**	16-10A1	1:200	Becton Dickinson
**CD86**	Y182A	1:200	Becton Dickinson
**CCR7**	4B12	1:200	Biolegend
**F4/80**	BM8	1:200	eBiosciences
**Foxp3**	FJK-16s	1:100	eBiosciences
**Gr-1**	RB6-8C5	1:200	Becton Dickinson
**I-A/I-E**	M5/114.15.2	1:600	BioLegend
**IFNγ**	XMG1.2	1:100	Biolegend
**TCRβ**	H57-597	1:200	Becton Dickinson

Supplementary Table 3: Primers used for real-time PCR

Gene	Forward primer	Reverse Primer
**Rplp0**	AGATTCGGGATATGCTGTTGG	TCGGGTCCTAGACCAGTGTTC
**TGF-β1**	TGTGGAACTCTACCAGAAATATAGC	GAAAGCCCTGTATTCCGTCTC
**CCL21**	CCCTGCTTCAACCATTACATCTGC	CCTGCTGTCTCCTTCCTCATTCC
**IL-6**	TCTATACCACTTCACAAGTCGGA	GAATTGCCATTGCACAACTCTTT
**IL-1β**	GAAATGCCACCTTTTGACAGTG	CTGGATGCTCTCATCAGGACA
**TNFα**	CAGGCGGTGCCTATGTCTC	CGATCACCCCGAAGTTCAGTAG
**IL-10**	ACAGCCGGGAAGACAATAACT	GCAGCTCTAGGAGCATGTGG
**CCL2**	CTGGAGCATCCACGTGTTGG	CATTCCTTCTTGGGGTCAGC
**CCR7**	TGTACGAGTCGGTGTGCTTC	GGTAGGTATCCGTCATGGTCTTG
**COX-1**	TCCATCCACTCCCAGAGTCAT	GTAGCCCGTGCGAGTACAAT
**COX-2**	TGACCCCCAAGGCTCAAATA	CAGGTTCTCAGGGATGTGAGG
**PTGIS (h)**	GGGTCTCCTCGACTTCTCCT	CAGGCGACTTTTGACACTGC
**PGES (h)**	GGATGCCCTGAGACACGGA	AGAAAGGAGTAGACGAAGCCC
**PTGIS (m)**	GAATCCTGCGGTCCGGTG	TGTCACCGTGCTTTTCCTTCA
**PGES (m)**	GTCCCCGAGTTGAAGTCCAG	GCTCCACATCTGGGTCACTC
**VEGFR1**	TGGCTCTACGACCTTAGACTG	CAGGTTTGACTTGTCTGAGGTT
**VEGFR2**	TTTGGCAAATACAACCCTTCAGA	GCAGAAGATACTGTCACCACC
**VEGFR3**	CTGGCAAATGGTTACTCCATG A	ACAACCCGTGTGTCTTCACTG
